# How do urban public health services affect rural migrant women's fertility intentions? A study based on the Mobile Population Dynamics Monitoring Survey in China

**DOI:** 10.1186/s12913-023-09219-8

**Published:** 2023-03-07

**Authors:** He Jiang, Yanshuo Huang

**Affiliations:** 1 School of Public Adiministration and Law, Hunan Agricultural University, Changsha, 410000 China; 2 Business School of Xiangtan University, Xiangtan, 411100 China

**Keywords:** Public health services, Rural migrant women, Fertility intentions

## Abstract

Public health service is an important guarantee by the government to safeguard the health rights of rural migrant women. This not only concerns the health status of rural migrant women and their willingness to stay in the urban area but can also affect their fertility intention. This study systematically examined the impact of public health services on the fertility intentions of rural migrant women as well as the mechanisms, underlying these intentions based on the data from the 2018 China Migration Dynamics Monitoring Survey. Urban public health services, including health records management and health education, could effectively enhance the fertility intentions of rural migrant women. Furthermore, their health status and willingness to stay in urban areas were important mechanisms, by which, the public health services could influence the fertility intentions of rural migrant women. Additionally, urban public health services have a better effect on improving the fertility desire of rural migrant women who have no pregnancy experience, a low income level, and a short residence time in the inflow area. This study contributed to the examination and clarification of the policy effects of public health services on the fertility intentions of rural migrant women. Additionally, it also provided important evidence to support the government policies related to the optimization of the public health service system, improvement of the health status, citizenship, and fertility intentions of the rural migrant women, as well as the development of the uniform public health services.

## Introduction

With the comprehensive and in-depth development of industrialization, marketization, urbanization, and internationalization of China's economy and society, people's concept of fertility has undergone a major change. Women of childbearing age have continued to decline in their willingness to have children. The average number of children planned to have dropped from 1.76 in 2017 to 1.64 in 2021. The total fertility rate of the whole society has dropped sharply from 5.6 in 1970 to 1.06 in 2022, which is not only lower than the world average fertility level of 2.5 but also below the population replacement level of 2.1 and the international alert level of 1.5 [1, 2]. According to the bulletin of the National Bureau of Statistics in 2022, the net growth of China's population in 2021 will be -850,000, with a natural growth rate of -0.60. This is the first time that China's population has experienced negative growth since 1962. The situation of population growth in the country is very serious, which has triggered the anxiety of the whole society about "zero population growth," "negative growth," and the "low fertility trap" [3, 4]. What is more worrying is that the development of China's low fertility rate has the characteristics of social integrity, structure, and sustainability, and the trend of negative population growth in urban and rural areas continues to show and strengthen [5]. Many scholars have found that while the fertility rate of the population in all parts of the country has generally declined, the low fertility rate in urban areas has occurred earlier and developed faster, so the situation is more severe than that in underdeveloped rural areas, which makes the problem of the low fertility rate of the population in China worse [6, 7]. The National Bureau of Statistics found that in the process of China's economic and social transformation and development, a large number of rural people have moved between cities and rural areas for a long time. According to the Dynamic Monitoring Data of the Floating Population in 2018, women accounted for 48.59% of the total floating population in China, and women of childbearing age (aged 15–49) were close to 90%. According to the data of the "Seventh General Survey," China's floating population was 376 million in 2020, and the estimated number of rural migrant women of childbearing age was 125 million (National Bureau of Statistics, 2021).Such a large number of rural women of childbearing age have moved between urban and rural areas for a long time, and their lifestyle, conception, and behavior of childbearing are inevitably affected by the dual factors of urban and rural social structure. Their fertility intentions may be different from those of rural non-migrant women and may also be different from those of women living in urban areas [8]. Although scholars believe that rural migrant women's fertility rates will follow the same low fertility development process as women in general, how does the fertility intentions of rural migrant women change? The academic community is far from reaching consensus [9–12]. Therefore, the issue of rural migrant women's fertility intentions needs further theoretical discussion.

In order to cope with the severe problem of population fertility, Chinese governments at all levels have actively adjusted policies to stimulate residents' fertility intentions and increase the social fertility rate. However, the effect of the fertility policies adopted by governments at all levels, from "double single child" to "single child" to "comprehensive two child," is far lower than the policy expectation [13, 14]. In the report of the 19th National Congress of the Communist Party of China, the State Council emphasized "promoting the matching connection of fertility policies with relevant economic and social policies." The outline of the national "Fourteenth Five-Year Plan" also clearly requires "improving public service projects and strengthening the grass-roots public health system" to provide a good policy environment for residents' fertility and boost their fertility intentions. In 2021, the CPC Central Committee and the State Council further issued the Decision on Optimizing the Fertility Policy and Promoting the Long-term and Balanced Development of the Population, requiring the further opening of the three-child birth restriction and supporting the optimization of policy measures to encourage urban and rural residents to increase their fertility intentions. Many scholars have admitted that there have been structural changes in the fertility behavior of Chinese residents. At the same time we open the birth restriction, we need to strengthen the support of public service policies to help achieve the expected fertility goal of the policy [15]. China's public services have long implemented the urban–rural dual supply system, and it is difficult for rural migrant women to enjoy public health services with equal opportunities for citizens in the cities where they flow into China. It was not until 2013 that the public health service policy for the migrant population was launched. In 2016, the Central Committee of the Communist Party of China issued the Outline of the Healthy China "2030" Plan to vigorously promote the equalization of basic public health services for the migrant population. We will greatly improve the public health security of the rural migrant population [16]. It has an important impact on the health of the rural migrant population, labor supply, feasible ability, urban adaptation, and quality of life [17–19]. As an important public service policy in urban and rural areas, how does urban public health service affect the fertility intentions of urban and rural residents, especially rural migrant women? What mechanism will affect the fertility intentions of rural migrant women? At present, these issues have received little attention and need further discussion.

Therefore, the main purpose of this paper is to discuss the internal mechanisms and main effects of urban public health services on rural migrant women's fertility intentions.The following parts of this paper are arranged as follows: The second part is literature review and research hypothesis, which is mainly to sort out relevant literature results and put forward theoretical assumptions; The third part is the research design, mainly including data sources, variable selection and description, and empirical regression model setting. The fourth part is the analysis of empirical results. Using the data from the 2018 China Mobile Population Monitoring Survey, the mechanism and effect of urban public health services on rural women's fertility intentions are determined; The fifth part is the conclusion and discussion.

## Literature review and research hypothesis

### Literature review

The scale of rural women's mobility in China is huge, and because of the barrier between the urban–rural dual registered residence system and the unbalanced development, various economic and noneconomic costs in urban and rural areas are growing rapidly, and the pressure on urban and rural survival and development is increasing. Rural migrant women can neither enter the city nor return to the countryside for a long time. They have repeatedly demonstrated "amphibious" mobility between urban and rural areas [20], and their fertility concept has always fluctuated between rural socialization and urban socialization. Their fertility intention has always been dynamically evolving under the influence of the urban–rural dual economy, society, life, values, etc [21–24]. Scholars generally believe that the change in rural migrant women's fertility intentions is affected by both the common determinants of population fertility and the personality factors of rural migrant women.

Scholars in mainstream demography, sociology, economics, and other fields believe that there are many common factors in population fertility decision-making. Economic and social development and the growth of the means of living affect the macro-cyclical changes of fertility intentions [6, 25]. The social status of the floating population, the concept of marriage and childbearing culture, the level of income and wealth, the cost of childbearing, the value of children, education and occupation, the level of physical health, age, and the sex of children The preference for quantity and quality directly determines the fertility choice [26–34]. The government's family planning and public service policies have a significant regulatory effect on fertility intentions [35–37]. A large number of theoretical and empirical studies in economics, sociology, and demography also found that women's childbearing and work conflict led to women's employment informalization, employment interruption, employer discrimination, workplace unfairness, depreciation of human capital, etc., resulting in complex "fertility punishment" [38], and in different stages of social development, women's "fertility punishment" effects at different stages of life were different, leading to the increase of women's fertility costs and affecting their fertility intentions. It is estimated that the "fertility punishment" effect of British women is between 4.4% and 20% [38], and the "fertility punishment" of American women is about 5% to 20% [39], while the "fertility punishment" of Chinese women leads to a decrease of women's hourly wage by 7% to 18% [40]. Such a huge "fertility punishment" will inevitably affect women's fertility intentions.However, some researchers discovered that the in-depth evolution of social and cultural systems, particularly the development of social low fertility cultural systems, weakened women's "fertility punishment," and thus the impact of "fertility punishment" on women's fertility intentions be weakened as well [41].

Rural migrant women move between urban and rural areas, and their fertility intentions are also affected by migrant personality factors. Scholars have grasped the particularity of migrant women's inevitable mobility, put forward different interpretation theories, and analyzed the impact of the special factors of mobility on migrant women's fertility intentions. According to the theory of floating birth interruption, rural floating women have many life, production, physiology, and psychology interruption effects, such as spouse selection interruption, marital life interruption, and reproductive physiology and psychology interruption, due to the regional mobility between urban and rural areas, and their fertility intention has changed [42–44]. Potter & Kobrin found that when the population moved from Mexico to the United States, the fertility intention had a significant effect on the interruption of the couple's life. The couple's separation for 4–7 months led to a 15% reduction in the fertility possibility in the second year, and the couple's separation for 8–12 months led to a 32% reduction in the fertility possibility in the second year [43]. Some scholars have studied further and found that the effect of birth interruption caused by mobility will change with the length of mobility. And in areas with a low contraceptive rate and a high fertility rate, the effect of birth interruption caused by mobility is more obvious, and the fertility rate decreases more [45]. However, some scholars of interruption theory believe that the internal mechanism of the birth interruption effect of migrant women may be more complex because migrant women, as individuals with reproductive decision-making, can have rational planning for the reproductive behavior of their individual life cycle and will break the flow or change the relationship of the reproductive period according to their individual reproductive needs, so as to prevent sexual reproduction before the flow or carry out "compensation" reproduction after the survival and development of the flow place is stable. Therefore, migrant women do not have a unique effect of migrant birth interruption that reduces their desire to have children throughout their lives [46, 47].

Scholars of the social adaptation theory of migrant childbearing believe that the rural–urban regional mobility of rural migrant women will be jointly affected by the economic and social development and cultural norms of the rural and urban areas where they flow out. Compared with rural society, the inherent modernity of cities makes urban production mode, life mode, social structure, cultural characteristics, and resource allocation completely different [48]. When rural migrant women enter the city, they feel the strong impact of urban citizens' ideas, behavior habits, lifestyle, workplace competition, education, medical care, residence, and social interaction and have to readjust and allocate their energy, time, and resources and make necessary adjustments to their physiology and psychology so as to enhance the adaptability of urban social survival and development. With the growth of urban life, rural migrant women gradually adapt to urban society, economy, values, lifestyle, and cultural customs, while rural society, economy, values, lifestyle, and cultural customs gradually fade away, resulting in the transformation of fertility concepts and practices from traditional to modern [12, 49, 50]. The urbanization and modernization of rural migrant women's fertility concepts have weakened the impact of rural local customs and traditional fertility concepts on fertility intentions. Even after controlling the flow interruption effect or selective effect of fertility, it still reduces the fertility intentions of rural migrant women, weakens the fertility preference of boys, prefers fewer and better births, and achieves quality-quantity substitution [23], which has a significant social adaptation effect on migrant fertility [44, 51]. Fertility intention was also significantly negatively affected [10]. However, as rural migrant women's urban social adaptation has increased, their pressure for survival and development has decreased, their sense of happiness has increased, and the fertility compensation effect has occurred, potentially leading to an increase in fertility intentions and adaptability [12].

As a common factor influencing women's fertility, public health services have a significant impact on this. On the one hand, some scholars believe that public medical and health services will change people's conception of fertility and reduce their willingness to procreate. Research shows that the underdeveloped public health services in Europe in the nineteenth century led to high fertility in Europe [52]. Jiang et al. found that the public medical subsidies in China's planned economy period directly reduced people's desire to have children [53]; On the other hand, public medical and health services will improve women's fertility intentions because they will improve women's fertility safety [54], which will also improve women's health and happiness levels. Women's desire for a better life will increase, and their fertility intentions may be higher [55]. Kong Zeyu found that women's self-satisfaction with medical and health services would significantly increase their willingness to bear [56]. However, some scholars also found that even if public medical and health services can improve people's health levels, they may not necessarily improve fertility intentions [37, 57]. On the other hand, the improvement of public medical and health services will improve the health level of infants and children, reduce the risk of infant and child death, increase fertility certainty, and possibly reduce fertility intentions [58, 59]. Finally, public health services will directly reduce the cost of women's fertility, and women's fertility intentions change accordingly.

It can be seen that the existing research has carried out fruitful multi-dimensional research on the floating population's fertility intentions, deeply explored the internal commonality and personality determinants of the floating population's fertility, and examined the impact of public health services on fertility intentions, but the research still has some deficiencies. First of all, China has long adopted the urban–rural dual system. The sharing of public health services between urban and rural residents is not equal. The majority of rural migrant women are "amphibious" in urban and rural areas. In fact, the public health services they enjoy are a mixture of leading rural public health services and limited urban public health services, which is unique in China. The existing research did not pay close attention to the urban–rural dual complexity and "amphibious" uniqueness of the public health service for migrant women with Chinese characteristics, and did not analyze the mechanism and effect of its impact on the fertility intentions of rural migrant women; Secondly, although governments at all levels "attach great importance to and accelerate the coordinated development of urban and rural public health services and the equalization of public health services," accelerate the process of equalization of public health services for the floating population, and rural migrant women have the opportunity to access urban public health services, there are differences in the needs and access levels of different rural migrant women groups for urban public health services. There is no empirical confirmation of the impact of urban public health services on the fertility intentions of different rural migrant women groups in China. These deficiencies need to be discussed in depth.

### Research hypothesis

The so-called "fertility intentions" refers to the expression of the individual's fertility intentions based on the preference for children and taking into account various restrictions, including the number of children expected to have children, gender, fertility time, and interval [60]. According to the above, rural migrant women, as individuals with fertility decision-making, will have their fertility change due to both the common determinants of population fertility and the individual factors of rural migrant women. From the perspective of common determinants, rural migrant women with different age, education, health, occupation, and concept endowments will weigh the relationship between children's value and fertility cost, dynamically adjust fertility strategies, and reasonably arrange fertility behavior in order to improve their own or family fertility efficiency; From the perspective of personality determinants, the majority of rural migrant women will face many interruptions in life, production, physiology, and psychology due to the "amphibious" flow in urban and rural areas. As a result, they will interrupt the flow or change the relationship during the reproductive period based on their individual reproductive needs, taking advantage of the opportunity to prevent sexual reproduction before the flow or "compensate" for fertility after the survival and development of the inflow area is stable; In addition, rural migrant women are facing a strong impact on their survival and development in urban social employment, education, medical treatment, residence, and social interaction. They have to readjust and allocate their energy, time, and resources and make necessary adjustments to their physiology and psychology to enhance their adaptability to urban social survival and development, and then adjust their fertility strategies accordingly.

To explore the impact of urban public health services on rural migrant women's fertility, the key is to clarify the internal logic of urban public health services affecting rural migrant women, which needs to be traced back to its source and analyzed from the basic connotation of public health services. The public health service, as a public product with the core goal of safeguarding people's health rights and interests, provides various forms of information dissemination and targeted behavioral intervention by providing public health education, establishing health record, and providing other health services. On the one hand, by promoting the efficiency and fairness of the supply of medical and health services in the whole society, reducing the cost burden of rural migrant women's use of medical and health services, alleviating the pressure of rural migrant women's survival and development on themselves and their families due to childbirth, and thus improving the level of rural migrant women's fertility intentions, on the other hand, it can help rural migrant women master health care knowledge, establish health concepts, change unhealthy lifestyles, develop good health habits, and ultimately improve their health level. In addition, since the launch of the National Basic Public Health Service Project in 2009, governments at all levels have attached great importance to and accelerated the coordinated urban and rural development of public health and the equalization of public health services. Rural migrant women and the local registered resident population can enjoy the government's public health services equally. This kind of institutional equity guarantee helps to enhance the sense of identity and belonging of rural migrant women to the local area, improve their adaptability to urban social survival and development, make them more willing to integrate into the inflow area and make long-term living arrangements, and reduce their reproductive burden and uncertainty accordingly.

Therefore, with regard to the relationship between urban public health services and rural migrant women's fertility intentions, on the one hand, urban public health services, through various forms of health service guarantees, make rural migrant women's fertility cost relatively lower and adjust the balance between their children's value and fertility cost accordingly, which may change their fertility intentions; [61]; Second, urban public health services can enhance rural migrant women's fertility and help them avoid their reproductive health risks by enriching their health knowledge, improving their health literacy, and finally changing their fertility intentions [18, 19]; Third, urban public health services can enhance rural migrant women's urban social adaptability, enhance their willingness to stay in the city for a long time, reduce their mobility costs, and reduce their fertility burden and uncertainty. Their fertility intentions could be modified [62]. Based on the above analysis, this paper proposes the following research hypotheses:

1. Urban public health services directly affect the fertility intentions of rural migrant women.
2. Urban public health services affect the fertility intentions of rural migrant women through the health mechanism.
3. Urban public health services affect the fertility intentions of rural migrant women through the willingness to stay mechanism.

**Fig. 1:**
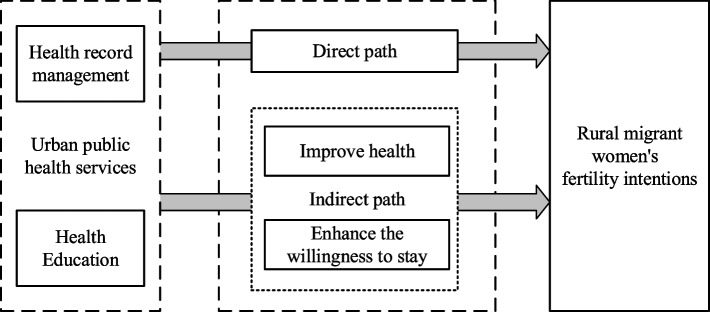
Logic of urban public health services, affecting the fertility intentions of rural migrant women

The logic of urban public health services, affecting the fertility intentions of rural migrant women is shown in Fig. 1.

## Research design

### Data sources

The empirical data presented in this article was taken from the China 2018 National Migrants Dynamic Survey (CMDS), which was organized and implemented by the National Health Commission using a hierarchical, multi-stage, and scale proportional Probability Proportionate to Size Sampling (PPS) method. This survey covered 31 provinces (autonomous regions and municipalities directly under the Central Government) and the Xinjiang Production and Construction Corps and analyzed the public health services and the fertility intentions, health, residence willingness, employment, and other information of the migrating population. All of these were consistent with authoritative, scientific, large-scale, and targeted characteristics, and the research theme of the study. Since this focused on the fertility intentions of rural migrant women, only the women between the ages of 15 and 49, who were in their occasional childbearing age and resided in the urban area for six months or more, including those having an agricultural hukou, were included in this study. After screening, a total of 34,092 samples were included in this study.

### Selection of variable and description

#### Interpreted variables 

This study was interpreted as "fertility intention". The variables were taken from the CMDS 2018 questionnaire. For the question, "Do you have any plans to have children this year and next?" The answers "yes", "no", and "did not think well" respectively indicated that the respondents were willing to have children this or next year, did not have the willingness to have children in this or next year, and did not know their willingness to have children this or next year. These fertility intentions were measured as a binary variable, if the respondent selected "Yes", the variable was assigned to 1, while the value of "No" or "did not think well" is 0. Based on this, a total of 3623 samples were assigned 1, accounting for 10.63% of the valid samples included in this study.

#### Explanatory variables

Public health education and implementing the health file management in migrating populations were considered the key construction contents of urban public health services issued by The National Health and Family Planning Commission in the 2013 "Pilot Work Plan for Equalization of Basic Public Services for Health and Family Planning of Floating Populations". In this study, "public health education" and "health record management" were set as the core explanatory variables [19].

##### Public health education

For the question in CMDS 2018 questionnaire, "Have you received health education in your current community/workplace in the past year?", the public health education items, received by each respondent included "occupational disease prevention", "infectious disease prevention, and treatment", "reproductive health and maternal and child health", "chronic disease prevention and treatment", "mental health", "public emergency self-help", and "other aspects", scored from 0 to 7 respectively [35]. According to CMDS 2017 data, "other aspects", included health education on smoking control. On average, each migrant female received at least two public health education items, and 18.56% of rural migrant women did not receive any public health education.

##### Health record management

For the question in CMDS 2018 questionnaire, "Has the resident health file been established for you", the answers received included "Yes, has been established", "Not built, not heard", "Not built, but heard of", and "Do not know". If the respondent selected "Yes, has been established", the variable was assigned 1, while the other options were assigned as 0. The study variable received a total of 10,220 answers assigned 1, accounting for 29.98% of the total respondents.

#### Covariates

In this study, a series of covariates, reflecting the characteristics of rural migrant women, spouses, families, mobility, and inflow areas were empirically controlled. Among these, the individual characteristics were reflected in controlling the age, ethnicity, years of education, marital status, political status, employment status, participation in urban medical insurance, and the number of children of the rural migrant women. The family characteristics were reflected in controlling the nature of spousal hukou, family ethnic characteristics, family income level, and family housing burden of the rural migrant women. The mobility characteristics were reflected in variables, such as controlling the movement range of rural migrant women and the time duration they spent in the newly migrated place. The newly migrated places by the rural migrant women were characterized as the provincial areas.

#### Mechanism of variables

The theoretical part demonstrated the effects of urban public health services on the fertility intention of rural migrant women to have children via health mechanisms, labor intensity mechanisms, and residence willingness mechanisms. For the health variable in the questionnaire, "In the last year, have you been sick (injured) or unwell?", the answers included "Yes, the most recent occurred within two weeks", "Yes, the most recent occurred two weeks ago", and "No". For the first two answers, the assigned variable was 0, while for the last answer, it was 1. The residency willingness variable was measured from the question "If you intend to stay in the local area, how long do you expect to stay there?". If the respondent was willing to stay for 5 years or more, the variable was assigned as 1, while it was 0 for any other answer. The specific definitions and descriptive statistics of each major variable are listed in Table 1.

**Table 1:** Definitions and descriptive statistics of main variables

variable		definition	average value	standard deviation
Interpreted variables	fertility intentions	Whether the respondents have any plans to have children this year and next: yes = 1, no = 0	0.106	0.308
Explanatory variables	Health records	Whether the respondent has established a local health record: yes = 1, no = 0	0.299	0.458
	Health education	Number of health education received by respondents in their current villages (residences) in one year (items)	2.075	1.827
Covariates	age	Respondent's age (years)	34.767	7.502
	Educational attainment	Respondents' academic qualifications: high school/technical secondary school and above = 1, otherwise = 0	0.318	0.466
	Political identity	Whether the interviewee is a Member of the Communist Party: yes = 1, no = 0	0.023	0.149
	marital status	Marital status of respondents: first marriage = 1, remarriage = 0	0.972	0.164
	Employment status	Whether the respondents had worked for more than one hour with income in the week before May Day: yes = 1, no = 0	0.74	0.439
	Whether to participate in the town health insurance	Whether the respondents participated in urban resident medical insurance, urban workers' medical insurance, or publicly funded medical care: yes = 1, no = 0	0.224	0.417
	Number of children	Number of biological children of the respondents	1.505	0.726
	The nature of the spouse's household registration	Whether the interviewee's spouse is an agricultural household registration: yes = 1, no = 0	0.098	0.297
	Ethnic characteristics of the family	Whether the respondent and any of his or her spouses are ethnic minorities: yes = 1, no = 0	0.116	0.319
	Household income level	The natural logarithm of the average monthly gross household income of the respondents	10.213	0.166
	Family housing burden	Respondent households spend an average monthly housing expenditure as a percentage of total expenditure (%)	21.6	20.5
	Range of flow	Whether the respondents were on inter-provincial mobility: yes = 1, no = 0	0.520	0.499
	The length of residence in the place of inflow	Length of residence in the place of inflow (years)	6.492	5.383
Mechanism variables	Health status	Whether the respondent was sick (injured) or unwell within one year: yes = 0, no = 1	0.878	0.327
	Willingness to stay	Whether the respondent intends to stay in the local area for 5 years or more: yes = 1, no = 0	0.634	0.482

### Empirical regression model setting

#### Benchmark regression model

In this study, a logit model was used to investigate the impact of urban public health services on the fertility intentions of rural migrant women. Due to the binary nature of explanatory variables, the model expression was calculated as given in Eq. (1).

1 $${FD}_{i}={\beta }_{0}+ {\beta }_{1}{PHS}_{i}+ {\beta }_{2}Control+ {\varepsilon }_{i}$$

where ${FD}_{i}$ indicated the willingness of rural migrant women to have children, ${PHS}_{i}$ indicated the public health services, and ${Control}_{i}$ indicated the control factors, such as personal endowment characteristics, family characteristics, and mobility characteristics of rural migrant women.${\beta }_{0}$, ${\beta }_{1}$, ${\beta }_{2}$ were the estimated coefficients in this study, among which, ${\beta }_{1}$ reflected the effects of public health services on the fertility intention of rural migrant women, and ${\varepsilon }_{1}$ indicated the random disturbance items.

#### Sample selectivity bias

The rural migrant women did not have random access to the public health services, relying on their self-selection of public health service needs to some extent. Therefore, the heterogeneity within this group would result in a large selective bias in the benchmark model estimates. In this regard, Rosenbaum & Rubin recommended the use of propensity score matching (PSM) to correct the sample selectivity bias in order to achieve an effect similar to that of the randomized trials [63]. However, Diamond & Sekhon argued that the PSM method did not assess the equilibrium of covariates well and that the estimated PSM was not reliable [64]. In order to solve this issue, Hainmueller & Xu proposed a solution as the entropy equilibrium matching method [65], which had many advantages over PSM. First, it could ensure that the processing and control groups were balanced in the sample characteristics and retained the useful information of all samples. Second, in the second phase of estimation, the model setting was more flexible. Third, the average difference test outcomes of covariates matched by this method were more reliable.

The basic idea of the entropy equilibrium matching method was constructing a binary dummy variable of the rural migrant women's access to the urban public health services, ${PHS}_{i}=\left\{\mathrm{0,1}\right\}$ which ${PHS}_{i}=0$ represent control groups and processing groups, respectively. By introducing the $j$ covariate and construction matrix, the sample average effect expression was obtained, which is given in Eq. (2).

2 $$ATT=E\left[{Y}_{1}|PHS=1\right]-E\left[{Y}_{0}|PHS=1\right]$$

where $E\left[{Y}_{0}|PHS=1\right]$ represented a counterfactual result. The entropy equilibrium directly estimated the weights using the potential large equilibrium constraint set. The counterfactual results were obtained using Eq. (3).

3 $$E\left[{Y}_{0}|PHS=1\right]=\frac{\sum \left\{i|D=0\right\} {Y}_{i}{w}_{i}}{\sum \left\{i|D=0\right\}{w}_{i}}$$

where ${w}_{i}$ was the entropy equilibrium weight selected using the control group. This study selected these weights using Eq. (4), which minimized the entropy distance metric.

4 $$\underset{{w}_{i}}{\mathrm{min}}H\left(w\right)=\sum_{\left\{i|D=0\right\}}{w}_{i}\mathrm{log}\left({w}_{i}\right./\left.{q}_{i}\right)$$

Equation (4) obeyed both Eqs. (5, 6, 7) for the averaging and normative constraints.

5 $$\begin{array}{cc}\sum\limits_{\left\{i\vert D=0\right\}}w_ic_{ri}\left(X_i\right)=m_r,r\epsilon1,&R\end{array}$$

6 $$\sum_{\left\{i|D=0\right\}}{w}_{i}=1$$

7 $${w}_{i}\ge 0,\left\{i|D=0\right\}$$

where $${q}_{i}$$ was the benchmark weight, $${c}_{ri}\left({X}_{i}\right)={m}_{r}$$$${c}_{ri}\left({X}_{i}\right)={m}_{r}$$ 1 was applied as a set of R equilibrium constraints on the covariate moment of the weighted control group. This step first weights the covariates in the scheme using Eq. (5) to impose the constraints on each covariate. The constraints generally included first-order moments (mean), second-order moments (variance), and third-order moments (skewness). This ensured that the covariate distribution moments of the reweighted control and processing groups were consistent.

Using these equilibria, normative and non-negative constraints, the entropy equilibrium method minimized the entropy distance between $W$ and benchmark weight vector in Eq. (4) by searching for and selecting a set of unit weights$W={\left\{{w}_{i}, ,{w}_{n}\right\}}^{T}$. Finally, this weight was used to perform a weighted least squares regression analysis on the effects of urban public health services on the fertility intentions of rural migrant women [42].

## Analysis of empirical results

### Analysis of baseline regression results

This study first analyzed the effects of urban public health services on the fertility intentions of rural migrant women using logit regression. The regression results of the health records and effects of health education on the fertility intention of rural migrant women are listed in Table 2(a) and (c). Given that the logit regression was a nonlinear model, the obtained parameters were not the true marginal effects of the indicators. The marginal effects of health records and health education, affecting the fertility intention of rural migrant women are also listed in Table 2(b) and (d). It can be seen that the participation of rural migrant women in health records increased their fertility intentions significantly and also enhanced their probability of planning to have children in the next two years by 1.6%. Each additional health education, received by the rural migrant women increased their fertility intentions by increasing their probability of planning to have children in the next two years by 0.1%. These results suggested that the urban public health services were effective in increasing the fertility intentions of rural migrant women, thereby confirming Hypothesis 1.

**Table 2:** Impact of the urban public health services on the fertility intentions of rural migrant women

Variables	Health Record Management		Health Education	
	(a) Logit	(b) dF/dx	(c) Logit	(d) dF/dx
Public Health Services	0.252^a^	0.016^a^	0.022^c^	0.001^c^
	(0.049)	(0.003)	(0.012)	(0.001)
Age	-0.101^a^	-0.006^a^	-0.101^a^	-0.006^a^
	(0.004)	(0.000)	(0.004)	(0.000)
Education level	-0.06	-0.004	-0.057	-0.004
	(0.05)	(0.003)	(0.05)	(0.003)
Political Identity	0.264^b^	0.017^b^	0.268^b^	0.017^b^
	(0.11)	(0.007)	(0.11)	(0.007)
Marital Status	-0.919^a^	-0.057^a^	-0.909^a^	-0.057^a^
	(0.131)	(0.008)	(0.13)	(0.008)
Employment Status	-0.166^a^	-0.01^a^	-0.169^a^	-0.011^a^
	(0.051)	(0.003)	(0.051)	(0.003)
Whether to participate in urban Medical Insurance	-0.007	-0.000	-0.006	-0.000
	(0.055)	(0.004)	(0.055)	(0.004)
Number of children	-2.749^a^	-0.172^a^	-2.741^a^	-0.171^a^
	(0.05)	(0.003)	(0.05)	(0.003)
Nature of spouse's household registration	-0.139^b^	-0.009^b^	-0.146^b^	-0.009^b^
	(0.058)	(0.004)	(0.058)	(0.004)
Family Ethnic Identity	0.143^b^	0.009^b^	0.148^b^	0.009^b^
	(0.068)	(0.004)	(0.068)	(0.004)
Family income level	0.468^a^	0.029^a^	0.465^a^	0.029^a^
	(0.144)	(0.009)	(0.144)	(0.009)
Family housing burden	0.027	0.002	0.022	0.001
	(0.105)	(0.007)	(0.105)	(0.007)
Mobility range	-0.120^b^	-0.008^b^	-0.121^b^	-0.008^b^
	(0.054)	(0.003)	(0.054)	(0.003)
Length of residence in the influx	0.012^b^	0.001^b^	0.013^b^	0.001^b^
	(0.005)	(0.000)	(0.005)	(0.000)
Provincial effect	YES	YES	YES	YES
Observations	34,092		34,092	

① Values in parentheses are robust standard deviations; ② ^a^, ^b^, and ^c^ denote 1%, 5%, and 10% significance levels, respectively, as in the later section

Table 2 also shows that the fertility intentions of rural migrant women were not only affected by the urban public health services, but were also closely related to their personal, family, and mobility characteristics. For example, the characteristics, such as age, marital status, employment status, number of children, spouse’s household nature, and mobility range, greatly decreased the fertility intentions of rural migrant women, while other characteristics, such as political status, family ethnicity, family income level, and length of residence in the newly migrated place significantly increased the fertility intentions of rural migrant women. In particular, an increase in the age of rural migrant women per unit reduced their chances of having children in the next two years by 0.6%. As compared to the remarried rural migrant women, the first-married rural migrant women showed a 5.7% lower probability of having children. Employment reduced the probability of having children by about 1% in the next two years for rural migrant women. An increase in the number of children per unit decreased the probability of having children in the next two years by about 17.2% for the rural migrant women. Those with an agricultural spouse were also less likely to have children, showing a 0.9% decrease in the probability of having children in the next two years. As compared to the rural migrant women, who moved across provinces, the probability of having children in the next two years decreased by 0.8% among those, who moved within provinces. The rural migrant women with party membership showed a 1.7% increase in the probability of having children in the next two years. Either spouse in a household who was an ethnic minority can increase the probability of rural migrant women having children by 0.9% in the next two years. The fertility intentions of rural migrant women increases with increased household incomes, an increase in the unit household income level variable increased the probability of having children in the next two years by 2.9%. The increase in the time duration of migration increased the probability of having children in the next two years by 0.1%.

### Analysis of entropy equilibrium matching results

Receiving public health services by the rural migrant women was based on their "self-selection" to a certain extent and affected by the characteristics of individual covariates. A comparison of the fertility intentions of rural migrant women, seeking public health services, should be performed to analyze the systematic differences between the two groups. In order to alleviate the problem of between-group bias in the assessment of effectiveness, this study used the entropic equilibrium matching method to set constraints on the first-order moments (mean), second-order moments (variance), and third-order moments (skewness) for the covariates in the regression analysis. This ensured that the control and treatment group samples had equal-weighted means on each covariate, which helped in alleviating the problem of between-group bias in the assessment of effectiveness. As compared to the PSM, this method was more conducive to achieving a balanced distribution of variables among the groups and was less affected by the measurement error while achieving the accurate matching of samples. The binary nature of the core explanatory variables required the division of control and treatment groups. As a core explanatory variable in this study, “health records” was a binary variable, but public health education was not a binary variable. Therefore, this study, using different methodologies, made two attempts to adapt health education to a binary variable. First, the number of health education items received by the rural migrant women were used as the criteria for dividing the rural migrant women into treatment and control groups; those, having at least one health education, were assigned 1 and included in the treatment group, while those, lacking health education, were assigned 0 and included into a control group. This logic generated binary variables with 1, 2, 3, 4, 5, 6 health education items. Second, the rural migrant women were divided into treatment and control groups based on the contents of health education they received. For example, if the rural migrant women had received reproductive, maternal, and child health education, they were considered as the treatment group and assigned a value of 1, while those, lacking any of these education contents were considered as the control group and assigned a value of 0. The binary variables for each type of health education were generated using this logic. It was worth noting that, by adjusting the first-order matrix, the binary variables could be effectively matched [42], and no further second and third-order moment adjustments were required for such variables. Table 3 reports the Average value, variance and matching test results of main covariates before and after entropy equalization.

**Table 3:** Matching test for main covariates

Variables		Average value		Variance		Standardized Mean Difference SMD	Standard deviation t-test *p*-value
		Treatment group	Control group	Treatment group	Control group		
Age	Before entropy equilibrium	34.4	34.92	54.32	57.04	-0.000095	0.0000
	After entropy equilibrium	34.4	34.4	54.32	54.61	0	1
Education Level	Before entropy equilibrium	0.356	0.302	0.229	0.211	0.002333	0.0000
	After entropy equilibrium	0.356	0.356	0.229	0.229	0	1
Political Identity	Before entropy equilibrium	0.025	0.022	0.025	0.021	0.001187	0.0928
	After entropy equilibrium	0.025	0.025	0.025	0.025	0	1
Marital Status	Before entropy equilibrium	0.973	0.972	0.027	0.027	0.000366	0.9316
	After entropy equilibrium	0.973	0.973	0.027	0.027	0	1
Employment Status	Before entropy equilibrium	0.728	0.745	0.198	0.189	-0.000849	0.0000
	After entropy equilibrium	0.728	0.728	0.198	0.198	0	1
Whether to participate in town medical insurance	Before entropy equilibrium	0.242	0.216	0.184	0.169	0.001398	0.0000
	After entropy equilibrium	0.242	0.242	0.184	0.184	0	1
Number of children	Before entropy equilibrium	1.494	1.51	0.513	0.532	-0.000309	0.0000
	After entropy equilibrium	1.494	1.494	0.513	0.541	0	1
Nature of spouse's household registration	Before entropy equilibrium	0.847	0.882	0.129	0.104	-0.002684	0.0000
	After entropy equilibrium	0.847	0.847	0.129	0.129	0	1
Family Ethnic Identity	Before entropy equilibrium	0.121	0.113	0.107	0.101	0.000740	0.0467
	After entropy equilibrium	0.121	0.121	0.107	0.107	0	1
Family income level	Before entropy equilibrium	10.2	10.22	0.023	0.029	-0.008602	0.0000
	After entropy equilibrium	10.2	10.2	0.023	0.102	0	1
Family housing burden	Before entropy equilibrium	0.209	0.219	0.045	0.041	-0.002198	0.0000
	After entropy equilibrium	0.209	0.209	0.045	0.039	0	1
Mobility range	Before entropy equilibrium	0.441	0.554	0.247	-0.247	-0.004525	0.0000
	After entropy equilibrium	0.441	0.441	0.247	0.247	0	1
Length of residence in the influx	Before entropy equilibrium	6.485	6.495	28.17	29.33	-0.000004	0.0000
	After entropy equilibrium	6.485	6.485	28.17	29.15	0	1

$SMD=\left(\overline{{X }_{T}}-\overline{{X }_{C}} \right)/\sqrt{{S}_{T}^{2}\left({n}_{T}-1\right)+{S}_{C}^{2}\left({n}_{C}-1\right)/\left({n}_{T}+{n}_{C}-2\right)}$, where $\overline{{X }_{T}}$ and $\overline{{X }_{C}}$ denoted the means of each variable in the treatment and control groups, respectively, ${S}_{T}^{2}$ and ${S}_{C}^{2}$ denoted the variances of each variable in the treatment and control groups, respectively, and ${n}_{T}$ and ${n}_{C}$ denoted the sample size in the treatment and control groups, respectively

The data after entropy equilibrium matching were re-regressed. Table 4 enlists the results of entropy equilibrium matching estimates obtained from the effects of public health services on the fertility intentions, as well as the comparison of results before and after treatment. The magnitude and significance of the coefficients changed due to the use of entropic equilibrium matching. In particular, keeping the health records had a significant positive effect on the fertility intentions of rural migrant women and increased the coefficient of fertility intentions by 0.135 as compared to those, who did not keep health records. The health education variable had a significant and favorable impact on the fertility intentions of rural migrant women only if they had received at least four or more health education content. The effects of public health services on fertility intentions were related to the contents of health education received by the rural migrant women. Only the reproductive health, maternal, child health, and self-education for public emergencies affected the fertility intentions of rural migrant women, while the other aspects of health education had a significant negative effect on the fertility intentions of rural migrant women.

**Table 4:** Comparison of the urban public health services on the fertility intention after entropic equilibrium matching

Variables		Coefficient of public health service variables		Confidence interval at the 95% level	
		Before processing	After treatment	Before processing	After treatment
Health Record Management	Whether to establish a resident health record	0.252***	0.135***	[0.157,0.347]	[0.058,0.213]
		(0.049)	(0.039)		
Health Education	Have received at least 1 health education	0.042	-0.058	[-.075,0.159]	[-0.159,0.0439]
		0.06	0.052		
	Have received at least 2 health education	0.027	0.015	[-0.061,0.115]	[-0.058,0.089]
		0.045	0.037		
	Have received at least 3 health education	0.029	0.018	[-0.063,0.121]	[-0.058,0.094]
		0.047	0.039		
	Whether or not they have received at least 4 health education	0.118**	0.083*	[0.013,0.224]	[-0.004,0.169]
		0.054	0.044		
	Whether or not they have received at least 5 health education	0.147**	0.125	[0.023,0.269]	[-0.022,0.183]
		0.063	0.08		
	Have received at least 6 health education	0.214***	0.139**	[0.065,0.363]	[0.016,0.262]
		0.076	0.063		
	Have you received occupational disease prevention and control health education?	-0.044	-0.024	[-0.139,0.0518]	[-0.103,0.054]
		0.049	0.039		
	Have you received infectious disease control health education?	0.006	-0.021	[-0.084,0.096]	[-0.095,0.053]
		0.046	0.038		
	Have you received reproductive health and maternal and child health education?	0.245***	0.124***	[0.156,0.334]	[0.049,0.198]
		0.045	0.038		
	Have you received chronic disease prevention and control health education?	0.046	-0.009	[-0.051,0.143]	[-0.089,0.071]
		0.049	0.041		
	Have you received mental health health education?	0.094*	0.053	[-0.014,0.202]	[-0.035,0.141]
		0.055	0.045		
	Have you received self-help in public emergencies? health education?	0.071	0.077*	[-0.024,0.166]	[-0.001,0.155]
		0.048	0.039		
	Have you received other aspects of of health education?	-0.137**	-0.087*	[-0.261,-0.013]	[-0.189,0.014]
		0.063	0.052		

① Values in parentheses are robust standard deviations; ② ***, **, and * denote 1%, 5%, and 10% significance levels, respectively, as in the later section

### Mechanism test

For investigating the in-depth mechanism of the effect of urban public health services on the fertility intentions of rural migrant women and verifying the existence of the theoretical transmission channel between the health status and fertility intentions, the test models were established, which are given in Eqs. (8) and (9).

8 $${mediator}_{i}= {a}_{0}+ {a}_{1 }{PHS}_{i}+ {a}_{2}Control+ {\varepsilon }_{1}$$

9 $${FD}_{i}={\gamma }_{0}+{\gamma }_{1}{mediator}_{i}+{\gamma }_{2}{PHS}_{i}+{\gamma }_{3}Control+{\epsilon }_{2}$$

where ${mediator}_{i}$ was the mediating variable. This study chose health status and willingness to describe the results based on the theoretical analysis. The specific test steps were as follows. First, a model was used to see if there was a positive promoting effect on the correlation between the urban public health services and mediating variable (8). Based on its coefficient being significantly positive, the mediating variable was introduced into the basic empirical model (1) to obtain model (9) and determine if it met the criteria for a mediating mechanism based on its coefficient and significance.

The results of the mediating mechanism test are listed in Tables 5 and 6, where Table 5 enlists the results of the health status mediating mechanism test and Table 6 shows the results of the willingness to stay mediating mechanism test. In Table 5, the results of urban public health services (health record management) show that the health level of rural migrant women improved by 20.2% due to health records. The coefficient of the effect of urban public health services (health record management) on the fertility intentions of rural migrant women after the introduction of health status was 24.7%, which was lower than the baseline regression coefficient of 25.2% without the introduction of health status, thereby passing the mediating effect test. The results of urban public health services (health education) showed that the health level of rural migrant women improved by 5.7% through health education. The coefficient of the urban public health services (health education) effects on the fertility intentions of rural migrant women was 2.1% after the introduction of health status. This was lower than the baseline regression coefficient of 2.2% without the introduction of health status, thereby passing the mediating effect test. These findings showed that the improvement of health status was an important influencing mechanism of the urban public health services to increase the fertility intentions of rural migrant women.

**Table 5:** Mechanism test and health status of the rural migrant women

VARIABLES	Health Record Management		Health Education	
	Health Status	Intention to have children	Health Status	Intention to have children
Health Status		0.243***		0.251***
		(0.073)		(0.073)
Health Record Management	0.202***	0.247***		
	(0.040)	(0.049)		
Health Education			0.057***	0.021*
			(0.010)	(0.012)
Control variable	YES	YES	YES	YES
Provincial effect	YES	YES	YES	YES
Observations	34,092	34,092	34,092	34,092

① Values in parentheses are robust standard deviations; ② ***, **, and * denote 1%, 5%, and 10% significance levels, respectively, as in the later section

**Table 6:** Mechanism test and willingness to stay in the rural migrant women

VARIABLES	Health Record Management		Health Education	
	Willingness to stay	Intention to have children	Willingness to stay	Intention to have children
Willingness to stay		0.177**		0.193***
		(0.070)		(0.070)
Health Record Management	0.336***	0.246***		
	(0.039)	(0.049)		
Health Education			0.018*	0.022*
			(0.009)	(0.012)
Control variable	YES	YES	YES	YES
Provincial effect	YES	YES	YES	YES
Observations	34,092	34,092	34,092	34,092

① Values in parentheses are robust standard deviations; ② ***, **, and * denote 1%, 5%, and 10% significance levels, respectively, as in the later section

Table 6 shows the result of the intermediary mechanism test for the residency intention of rural migrant women. The health records could increase their residency intention by 33.6% after introducing the residency intention variable. The coefficient of the effects of health records on the fertility intentions of rural migrant women was 24.6%, which was lower than the baseline regression result of 25.2%, thereby passing the mediating effect test. Health education could increase the stay intention of rural migrant women by 1.8%, and the coefficient of the effect of health education on rural migrant women’s intention to have children was 2.2% after the introduction of the intention to stay variable. This was not significant in comparison to the decrease in baseline regression coefficient due to the retention of decimal places in the reported results, but it still passed the mediating effect test. As evidenced by the findings above, an increase in the willingness to stay was an important influencing mechanism of the urban public health services to improve the fertility intentions of rural migrant women.

### Further analysis

These studies confirmed the positive effects of urban public health services on the intentions of rural migrant women and verified their transmission pathways and mechanisms. The direct mechanisms of action and transmission pathways might be heterogeneously different between the various groups of rural migrant women due to the differences in the demand for and access to urban public health services. Therefore, this study deeply explained the heterogeneity in terms of pregnancy experience, income level, and the duration of inflow into residence.

These results showed that the women, who had previous pregnancy experience, were more likely to rely on their own experiences rather than urban public health services as compared to those, who had no previous pregnancy experience. The results of the heterogeneity analysis of pregnancy experience are listed in Table 7. The interaction term between health records and pregnancy experience (HR_PE) was -0.445, and that between health education and pregnancy experience (HE_PE) was -0.086, both of which were significant at the 1% level, indicating that women's pregnancy experience weakened the promotion effect of urban public health services on their fertility intention. The results of the heterogeneity analysis regarding the availability of pregnancy experience showed that urban public health services were more effective in increasing the fertility intentions of women with no pregnancy experience.

**Table 7:** The heterogeneity analysis about pregnancy experience

VARIABLES	Health Record Management	Health Education
Health Record Management	0.636***	
	(0.123)	
HRM_PE	-0.445***	
	(0.130)	
Health Education		0.095***
		(0.027)
HE_PE		-0.086***
		(0.028)
Control variable	YES	YES
Provincial effect	YES	YES
Observations	34,092	34,092

① Values in parentheses are robust standard deviations; ② ***, **, and * denote 1%, 5%, and 10% significance levels, respectively, as in the later section

Considering the impact of urban public health services on rural migrant women's fertility intentions from a cost perspective, then rural migrant women's high or low income would be an important moderating factor influencing urban public health services to promote their fertility intentions. This paper argues that with higher income, rural migrant women can maintain their health status through other channels and also face less life stress, thus the effect of urban public health services on the promotion of rural migrant women's fertility intentions will continue to decrease. The results of the heterogeneity analysis of income levels are shown in Table 8. The interaction term between health records and income level (HR_IL) was -0.82, which was significant at the 1% level, while the interaction term between health education and income level (HE_IL) was -0.047, but did not pass the significance test. The results of the heterogeneity analysis regarding income level showed that as the income level of rural migrant women increased, the promotion effect of health records on their fertility intentions gradually decreased, and the promotion effect of health education on their remaining intentions was not significantly affected. This is due to the fact that health records have an impact on health primarily at the level of health care cost savings, while health education has an impact on health primarily at the level of health awareness.

**Table 8:** The heterogeneity analysis about income level

VARIABLES	Health Record Management	Health Education
Health Record Management	8.638***	
	(2.988)	
HRM_IL	-0.820***	
	(0.292)	
Health Education		0.502
		(0.744)
HE_IL		-0.047
		(0.073)
Control variable	YES	YES
Provincial effect	YES	YES
Observations	34,092	34,092

① Values in parentheses are robust standard deviations; ② ***, **, and * denote 1%, 5%, and 10% significance levels, respectively, as in the later section

The length of time spent in the place of residence is an important influencing factor on rural migrant women's identity, environmental integration and fertility intentions. This paper argues that as the time of inflow to the place of residence increases, on the one hand, the long period of residence is conducive to the establishment of health records, and on the other hand, rural migrant women gradually have a stronger sense of belonging to the city and are more willing to accept health education. Thus the longer rural migrant women have been flowing into their place of residence, the stronger the effect of urban public health services in promoting their fertility intentions. The results of the heterogeneity analysis regarding the inflow time are shown in Table 9. The coefficient of the interaction term between health records and length of inflow to residence (HR_LIR) was 0.215 (significant at the 5% level), and the coefficient of the interaction term between health education and length of inflow to residence (HE_LIR) was 0.062 (significant at the 1% level). The results indicate that the promotion effect of urban public health services on rural migrant women's fertility intentions gradually increases as their time of inflow to their place of residence increases.

**Table 9:** The heterogeneity analysis about length of inflow to residence

VARIABLES	Health Record Management	Health Education
Health Record Management	0.161***	
	(0.060)	
HRM_LIR	0.215**	
	(0.084)	
Health Education		0.005
		(0.015)
HE_LIR		0.062***
		(0.018)
Control variable	Y	Y
Provincial effect	Y	Y
Observations	34,092	34,092

① Values in parentheses are robust standard deviations; ② ***, **, and * denote 1%, 5%, and 10% significance levels, respectively, as in the later section

## Conclusions and policy recommendations

This paper uses the 2018 CMDS data to study the impact of urban public health services on the fertility intentionsingness of rural migrant women and draws the following conclusions: (1) Urban public health services promote rural migrant women's fertility intentions through direct mechanisms, and the conclusion is still valid after using entropy equilibrium matching; (2) Through the mediation mechanisms of health status and willingness to stay, urban public health services promote the fertility intentions of rural migrant women; (3) Urban public health services have a better effect on improving the fertility desire of rural migrant women who have no pregnancy experience, a low income level, and a short residence time in the inflow area. The preceding study conducted an in-depth analysis of the mechanism and heterogeneity of urban public health services on rural migrant women's fertility intentions, providing reference empirical evidence for the government to formulate and improve the urban public health service system, improve the health status of rural migrant women and their level of citizenship and fertility intentions, and promote the homogenization and development of China's public health services.

The fertility behavior of rural migrant women is determined not only by the common factors of population fertility, such as age, gender, health, marriage and childbearing cultural concepts, income and wealth, fertility cost, child value, education, and occupation [25–34], but also by individual factors such as mobility interruption and social adaptation [42–44, 49–51]. Most scholars regard public health services as common determinants of women's fertility and point out that public health services may affect women's fertility intention asymmetrically by changing fertility concepts, reducing fertility costs, promoting health levels, reducing fertility risks, and improving satisfaction [52–59], while paying less attention to the impact of public health services on women's fertility from the perspective of personality determinants. Under China's urban–rural dual system, rural migrant women have been "amphibious" in urban and rural areas for a long time. In fact, the public health service they enjoy is a mixture of the leading rural public health service and the limited urban public health service. It has the urban–rural dual complexity and the "amphibious" uniqueness, and its internal logic that affects the fertility intentions of rural migrant women is inevitably unique. This study shows that the impact of urban public health services such as health education and health archives on the fertility intentions of rural migrant women is different from the logical relationship that public services such as social pension security squeeze out women's fertility by replacing traditional child support [66], but at the same time promote the fertility intentions of rural migrant women through the direct mechanism and the intermediary mechanism between health status and residence desire. Because of the long-term "amphibious" movement of rural migrant women in urban and rural areas, the rapid growth of various economic and non-economic costs in urban and rural areas, and the inability to effectively express their fertility intentions due to the huge pressure of urban and rural survival and development [21–24], while urban public health services such as health education and health archives, through various forms of information dissemination and targeted behavioral intervention, promote the efficiency and fairness of the supply of medical and health services in the whole society, On the one hand, it can reduce the cost burden of rural migrant women using medical and health services, alleviate the pressure on rural migrant women's own survival and development due to childbirth, and promote the expression of rural migrant women's fertility intentions; On the other hand, urban public health services can help rural migrant women avoid fertility risks by improving their health literacy and their health status, which can greatly reduce the economic and non-economic costs that rural migrant women bring to themselves because of fertility, thus helping to release their fertility intentions; In addition, urban public health services are usually provided locally in the inflow area, which helps to enhance rural migrant women's sense of identity and belonging to the local area and makes them more willing to integrate into the inflow area and make long-term living arrangements, which can greatly reduce the flow cost of rural migrant women, reduce their reproductive burden and uncertainty, and improve their fertility intentionsingness. The study also shows that urban public health services have a better effect on the fertility intentions of rural migrant women who have no pregnancy experience, a low income level, and a short residence time in the inflow area. This is because rural migrant women without pregnancy experience can further release their convergent fertility under the constraints of lack of fertility experience, high fertility risk uncertainty, and large fertility punishment by using urban public health services; In terms of income level, rural migrant women with higher incomes can not only maintain their health status through other channels but also face less pressure for survival and development. Therefore, the promotion effect of urban public health services on rural migrant women's fertility intentions continue to decline, and the effect will be more obvious for rural migrant women with lower income levels; In terms of residence time in the inflow area, the longer rural migrant women live in urban areas, the greater the impact of urban economic, social, and cultural life and values, the lower their reliance on their children, the narrower the demand space for childbearing, and the naturally smaller impact of urban public health services on their fertility intentions.

Based on the research findings in this study, the following policy recommendations were recommended. First, the supply of public health services should be strengthened. Even though urban public health services could effectively increase the fertility intentions of rural migrant women, the government should increase the financial investment in public health services, open up social financing channels, improve the level of public health services, broaden the basic coverage of public health services, and further eliminate the worries of rural migrant women about giving birth. Second, the quality of public health services should be improved. The quality and depth of health education services and flexible customization of the health education service curricula based on the acceptance ability and needs of rural migrant women should be improved while increasing the efficiency of health education services. Third, the construction of health file informatization should be accelerated. The inadequate construction of health file informatization in some remote areas and the existence of health file information barriers were not conducive to the timely access and application of the health file information, which greatly reduced the convenience of applying health files to the migrating population. Local governments should expedite the development of a health file management model, providing full coverage to both the urban and rural residents, high informatization, high accuracy, and weak barriers, as well as developing the homogenization and standardization of health file management services.

## Data Availability

The datasets used in the current study is publicly available from China Migration Population Service Center [https://www.chinaldrk.org.cn].
